# Kenyan MSM: no longer a hidden population

**DOI:** 10.1097/QAD.0000000000000928

**Published:** 2015-12-01

**Authors:** Eduard J. Sanders, Harold Jaffe, Helgar Musyoki, Nicolas Muraguri, Susan M. Graham

**Affiliations:** a Centre for Geographic Medicine Research – Coast, Kenya Medical Research Institute (KEMRI), Kilifi, Kenya; b Nuffield Department of Medicine, University of Oxford, Headington, UK; c Department of Global Health, Academic Medical Centre, University of Amsterdam, Amsterdam, the Netherlands; d Centers for Diseases Control and Prevention, Atlanta, Georgia, USA; e National AIDS and STI Control Programme (NASCOP); f Ministry of Health, Nairobi, Kenya; g Departments of Medicine, Global Health, and Epidemiology, University of Washington, Seattle, Washington, USA

In 2005, almost 25 years after the emergence of the HIV pandemic among MSM in the United States, the first substantial report of HIV and sexually transmitted infections (STIs) among a large group of MSM from Senegal was published in *AIDS* [1]. Although MSM received late recognition in the African HIV epidemic [2,3], Kenya was at the forefront in recognizing the vulnerabilities of this highly stigmatized population that feared legal authorities and had virtually no access to health services [4]. Numerous studies have since documented the elevated HIV/STI infection risks of African MSM, and donor responses have begun to focus on inclusion of MSM and their emerging organizations in HIV prevention and care programming in Africa [5]. Despite legal challenges and largely negative public debates [6], the Kenyan Ministry of Health and National AIDS and STI Control Programme has recognized that MSM are one of the key populations in need of urgent attention and have demonstrated their willingness to work with them [7].

This relatively supportive environment set the stage for recruitment of MSM into a cohort study investigating the feasibility of HIV-1 vaccine research on the Kenyan coast [8]. The Key Populations Cohort studies at the Kenya Medical Research Institute–Wellcome Trust Research Program in Kilifi, now in existence for 10 years, have reported the much higher HIV-1 incidence among MSM who had exclusive sex with men than in MSM who had sex with men and women [9]. In addition, numerous operational research studies based in this cohort have informed HIV prevention and care programming for MSM in Kenya and beyond [10–13]. In the past few years, HIV research with MSM in Kenya has expanded to several major cities. In Nairobi, over 1000 male sex workers, most of them MSM, have been engaged in research and provided with HIV care and prevention services [14,15], whereas counselling services targeting MSM have also been provided by the Liverpool Voluntary Counselling and Testing Programme [16]. In Kisumu, a Centers for Diseases Control and Prevention-funded study of a combination prevention and care programme for up to 700 MSM started enrolment in 2015, and an additional 100 MSM will be targeted for enrolment into an HIV Prevention Trials Network study of the feasibility of engaging and retaining MSM in research at sites in South Africa, Malawi and Kenya. As a result of this increased activity, researchers in Kenya have formed an MSM health research consortium, with the aim of improving healthcare for MSM and sharing findings with the Ministry of Health. Increasingly, research with MSM is informed by the views and planned with the support of Kenyan lesbian, gay, bisexual, and transgender groups. In addition to tackling health challenges, these LGBT groups and their leaders aim to address human rights challenges. Clearly, MSM in Kenya are no longer a hidden population.

The 10-year anniversary of the Key Populations cohort studies in Kilifi prompted the idea for this *Supplement*. This *Supplement* aims to recognize ongoing research with MSM in Kenya, stimulate ideas for new research and support efforts by communities themselves.

Three manuscripts in this *Supplement* focus on MSM enrolled in cohort studies in coastal Kenya and highlight the heterogeneity of HIV risk for MSM who sell sex to men, the challenges of reducing HIV risk for MSM through behavioural counselling and the risk of rape and physical assault faced by MSM.

In their study of MSM who sell sex, Smith *et al.* [17] report that the majority of study participants had both male and female sex partners. However, those with bisexual behaviour were not simply MSM who have sex with women; rather, they differed from exclusively homosexual men in important ways. Those with bisexual behaviour had a considerably lower HIV prevalence (8.3%) than those without female partners (50%) and had fewer behavioural risks for infection. Although bisexual male sex workers may potentially serve as a bridge for HIV transmission between MSM and women, Smith *et al.* make the important observation that because of the lower risk of HIV infection in these men, their role in bridging may have been overestimated in modelling projections of the Kenyan HIV epidemic [17].

Recognizing the high risk of HIV infection for MSM in coastal Kenya, Möller *et al.* [18] implemented risk reduction counselling for HIV-negative and HIV-positive MSM followed in a research clinic setting. Over the relatively short duration of follow-up, a median of 1.2 years, the authors found significant reductions in several sexual risk behaviours. Of particular interest, HIV-infected men decreased their rates of insertive anal intercourse, whereas uninfected men reduced their rates of receptive anal intercourse. However, the authors note several important study limitations. More than 25% of MSM initially enrolled made no follow-up visits. Furthermore, behavioural change was measured by self-report rather than by outcomes such as STI or HIV acquisition. Although further behaviour change research may be useful in these very high-risk MSM, biomedical interventions, including preexposure prophylaxis and treatment as prevention, are urgently needed.

Micheni *et al.* [19] report on the high rate of sexual, physical and verbal assaults on MSM in coastal Kenya. Rape incidence in MSM was similar to that reported in female sex workers (FSWs) and was associated with alcohol use, sex work and group sex. In contrast to the perpetrators of physical violence against FSW, who are usually regular sex partners, violence against MSM was often ‘opportunistic aggression,’ perpetrated by strangers. Although the authors call for structural interventions to protect both MSM and FSW, they note that although Kenyan HIV/AIDS policy recognizes the need to reduce violence against MSM and FSW, Kenyan law criminalizes these populations.

Three subsequent manuscripts in this *Supplement* address the need for interventions to support both the HIV-negative and the HIV-positive MSM in their uptake of and adherence to HIV prevention and care.

Harper *et al.* [20] investigated sexual and psychosocial resilience along with intrapersonal and interpersonal factors promoting resilience. Resilience has been defined as a process involving positive adaptation in the face of adversity and risk, and has received attention as a cornerstone of HIV prevention research for gay and bisexual men [21]. The study by Harper *et al.* [20] was noteworthy for its use of community-based participatory research methods to recruit over 500 young gay, bisexual and other MSM in Kisumu and surrounding areas. The young men surveyed were mainly HIV negative by self-report and 25% reported transactional sex. Among this group, problems including loneliness, lower perceived social support, reactions to trauma, low self-esteem, struggles with LGBT identity and internalized homonegativity adversely affected psychosocial well-being and uptake of preventive measures including condom use and HIV testing. Conversely, men who had higher perceived social support, higher self-esteem and acceptance of their gay or bisexual identity were more likely to take up preventive measures. The authors recommend that intervention components to foster resilience be incorporated to promote the success of HIV prevention programmes.

Secor *et al.* [22] have conducted a cross-sectional survey of mental health, including depression, substance abuse, HIV stigma and sexual minority stigma, among MSM participating in cohort studies on the Kenyan coast. The study population included many male sex workers, and almost half the participants were HIV positive. The main outcome measure was depression score, based on the Patient Health Questionnaire-9 instrument [23]. In this population, alcohol abuse, other substance abuse, sexual stigma and childhood or recent sexual abuse were all correlated, but did not differ by HIV status. Although higher scores on the sexual stigma scale used [24] were associated with higher depression scores, being married to a woman was associated with lower depression scores. Clearly, stigma reduction and comprehensive mental health services, recommended by the authors, may be needed to reduce barriers to care engagement in this and similar MSM populations.

Building successful programmes to help MSM adhere to HIV prevention and care recommendations is a challenge in Kenya, as in other African settings. Graham *et al.* [25] have taken a step in this direction by designing and pilot testing a tailored intervention to promote care engagement and antiretroviral therapy (ART) adherence among HIV-positive MSM. In-depth individual qualitative interviews with HIV-positive MSM, focus group discussions with experienced care providers and input from LGBT community groups and other relevant stakeholders formed the basis of the *Shikamana* (Kiswahili for ‘to form a bond or stick together’) intervention. The intervention combines a modified next-step counselling approach to promote men’s motivation and help them problem solve, with support from an ART-experienced peer trained to provide information, empathy and encouragement. Findings from the small pilot test conducted for this study show promise, as the intervention was found to be well tolerated, feasible and acceptable. The *Shikamana* intervention approach and data presented will be of great interest to other researchers and interventionists in designing programmes to promote appropriate, tailored services for African MSM living with HIV.

Two articles offer a systems approach to the vulnerabilities common among MSM. Van der Elst *et al.* [26] report that healthcare providers’ skills can be strengthened through training to improve HIV services for MSM. Earlier work demonstrated a reduction in homoprejudice [27] of healthcare providers who underwent an interactive eight-module training course now freely available online (www.marps-africa.org) [28]. The MSM sensitization training offers an opportunity for providers to change, and van der Elst *et al.* argue that it should be urgently offered at all medical training colleges and adopted by professional associations. Although over 1200 Kenyan healthcare providers, including over 570 providers in the public sector, have completed the online training in the past 5 years, the authors pose the larger structural problem that ‘HIVand health are not simply biomedical issues but social and political phenomena, which require huge efforts to change in society’.

Sanders *et al.* [29] report on the importance of targeted screening of patients for acute HIV infection (AHI). MSM who acquired HIV-1 in the cohort studies in Coastal Kenya informed the idea for this study, as many sought healthcare prior to seroconversion and were frequently treated for malaria [30]. AHI is the brief 2–3-week period following HIV-1 acquisition in which 40–90% of patients develop symptoms and signs, for which healthcare may be sought. Owing to the fact that HIV antibodies are not detectable during AHI, patients need evaluation with antigen or RNA-based tests, which are just becoming available to African HIV prevention and care programmes. Detection of AHI is important, as patients’ viral load is sharply elevated in this period, and prompt diagnosis permits counselling for sexual risk reduction and ART initiation. Sanders *et al.* [29] from four African research sites pooled data and showed that targeted screening of adults at risk for AHI using a simple algorithm based on seven features (age 18–29 years, fever, fatigue, diarrhoea, body pains, sore throat and genital ulcer disease) would substantially reduce the number of symptomatic HIV-1-seronegative patients requiring AHI testing. They recommend their risk score algorithm for use by HIV programmes in sub-Saharan Africa with capacity to test high-risk patients for AHI.

All of the work reported in this *Supplement* has been performed in a context wherein MSM are stigmatized and marginalized, and in which sex between two consenting males is illegal. For this reason, recruitment of MSM for research studies usually is done discreetly by word of mouth, through peer networks or with the help of LGBT organizations and other civil society groups. Men who are closeted or otherwise prefer to remain hidden are not reached easily by these approaches. Thus, none of these studies is truly population based. Although the study by Harper *et al.* [20], and, to a lesser extent, the study by Graham *et al.* [25], did reach men who were not sex workers, commercial sex work was commonly reported by the men included in these studies. Although this may be partly because of the high rates of unemployment in Kenya (estimated at 40%) [31] and frequent transactional sex among Kenyan men (approximately 3% of men aged 18–64 years reported ever having received money, gifts or favours in exchange for sex [32]), it is also a reflection of the focus of many HIV incidence cohorts, which often selectively target individuals at the highest risk for HIV-1 acquisition. Reaching out to MSM who are not sex workers, may not identify as gay, or may be more socially privileged would broaden our understanding of what it means to be an MSM in Kenya, and could serve to reduce stigma.

These *Supplement* studies provide suggestions for new directions in research and prevention in Kenya. First, if the HIV epidemic among MSM has less transmission impact on the general population than what was previously thought, then the modes of transmission study estimates for Kenya should be adjusted [33]. Kenya also has the potential to conduct a well designed population-based phylogenetic study to confirm the degree of mixing of HIVepidemics among MSM and the general population [34]. Such a study would require sequencing of samples from recently infected persons from the general population, MSM and other key populations, with detailed analysis of sequence clustering. Kenya collects blood for its periodic AIDS indicator survey and has many ongoing clinic-based cohorts as well as key population cohorts led by the MSM consortium and others with the requisite data on estimated date of HIV-1 infection, biological sex and route of transmission [35]. Second, reducing HIV-1 transmission in MSM populations in Kenya will need multiple interventions, including a ‘roll out’ of treatment as prevention and targeted promotion of PrEP among male sex workers and interested high-risk MSM, supported by the new WHO guidelines [36]. Increased coverage with biomedical prevention interventions should be combined with approaches outlined in this *Supplement*, including improved recognition of AHI, innovative peer support to promote medication uptake and adherence, engagement and collaboration with LGBT groups to strengthen social support and wider community stakeholder engagement, including healthcare workers trainings, to facilitate societal change. Third, the MSM health research consortium in Kenya has the opportunity to coordinate research efforts, align goals with both LGBT stakeholders and the Kenyan Ministry of Health and coordinate planning with partners on future intervention studies that hold the greatest promise to improve health for Kenya MSM.

## References

[1] Wade AS, Kane CT, Diallo PA, Diop AK, Gueye K, Mboup S (2005). HIV infection and sexually transmitted infections among men who have sex with men in Senegal. AIDS.

[2] Baral S, Sifakis F, Cleghorn F, Beyrer C (2007). Elevated risk for HIV infection among men who have sex with men in low- and middle-income countries 2000–2006: a systematic review. PLoS Med.

[3] Smith AD, Tapsoba P, Peshu N, Sanders EJ, Jaffe HW (2009). Men who have sex with men and HIV/AIDS in sub-Saharan Africa. Lancet.

[4] Onyango-Ouma W, Burungi H, Geibel S (2005). Understanding the HIV/STI risks and prevention needs of men who have sex with men in Nairobi, Kenya.

[5] Beyrer C, Sullivan PS, Sanchez J, Dowdy D, Altman D, Trapence G (2012). A call to action for comprehensive HIV services for men who have sex with men. Lancet.

[6] Nordling L (2015). African academics challenge homophobic laws. Nature.

[7] Bhattacharjee P, McClarty LM, Musyoki H, Anthony J, Kioko J, Kaosa S (2015). Monitoring HIV Prevention Programme Outcomes among Key Populations in Kenya: Findings from a National Survey. PLoS One.

[8] Sanders EJ, Graham SM, Okuku HS, van der Elst EM, Muhaari A, Davies A (2007). HIV-1 infection in high risk men who have sex with men in Mombasa, Kenya. AIDS.

[9] Sanders EJ, Okuku HS, Smith AD, Mwangome M, Wahome E, Fegan G (2013). High HIV-1 incidence, correlates of HIV-1 acquisition, and high viral loads following seroconversion among MSM. AIDS.

[10] Taegtmeyer M, Davies A, Mwangome M, van der Elst EM, Graham SM, Price MA (2013). Challenges in providing counselling to MSM in highly stigmatized contexts: results of a qualitative study from Kenya. PLoS One.

[11] Graham SM, Mugo P, Gichuru E, Thiong’o A, Macharia M, Okuku HS (2013). Adherence to antiretroviral therapy and clinical outcomes among young adults reporting high-risk sexual behavior, including men who have sex with men, in coastal Kenya. AIDS Behav.

[12] Van der Elst EM, Mbogua J, Operario D, Mutua G, Kuo C, Mugo P (2013). High acceptability of HIV pre-exposure prophylaxis but challenges in adherence and use: qualitative insights from a phase I trial of intermittent and daily PrEP in at-risk populations in Kenya. AIDS Behav.

[13] Sanders EJ, Wahome E, Okuku HS, Thiong’o AN, Smith AD, Duncan S (2014). Evaluation of WHO screening algorithm for the presumptive treatment of asymptomatic rectal gonorrhoea and chlamydia infections in at-risk MSM in Kenya. Sex Transm Infect.

[14] Muraguri N, Tun W, Okal J, Broz D, Raymond HF, Kellogg T (2015). HIV and STI prevalence and risk factors among male sex workers and other men who have sex with men in Nairobi, Kenya. J Acquir Immune Defic Syndr.

[15] McKinnon LR, Gakii G, Juno JA, Izulla P, Munyao J, Ireri N (2014). High HIV risk in a cohort of male sex workers from Nairobi, Kenya. Sex Transm Infect.

[16] Angala P, Parkinson A, Kilonzo N, Natecho A, Taegtmeyer M Men who have sex with men (MSM) as presented in VCT data in Kenya.

[17] Smith AD, Muhaari A, Agwanda C, Kowuor D, Van der Elst E, Davies A (2015). Heterosexual behaviours among men who sell sex to men in Coastal Kenya. AIDS.

[18] Möller LM, Stolte IG, Geskus RB, Okuku HS, Wahome E, Price MP (2015). Changes in sexual risk behavior among MSM participating in a research cohort in coastal Kenya. AIDS.

[19] Micheni M, Rogers SM, Wahome E, Darwinkel M, Van der Elst E, Gichuru E (2015). Risk of sexual, physical and verbal assaults on men who have sex with men and female sex workers in coastal Kenya. AIDS.

[20] Harper GW, Wade RM, Onyango DP, Abuor PA, Bauermeister JA, Odero WW (2015). Resilience among Gay/Bisexual Young Men in Western Kenya: Psychosocial and Sexual Health Outcomes. AIDS.

[21] Herrick AL, Stall R, Goldhammer H, Egan JE, Mayer KH (2014). Resilience as a research framework and as a cornerstone of prevention research for gay and bisexual men: theory and evidence. AIDS Behav.

[22] Secor AM, Wahome E, Micheni M, Rao D, Simoni JM, Sanders EJ (2015). Depression, Substance Abuse, and Stigma among Men Who Have Sex with Men in Coastal Kenya. AIDS.

[23] Kroenke K, Spitzer RL, Williams JB (2001). The PHQ-9: validity of a brief depression severity measure. J Gen Intern Med.

[24] Logie CH, Newman PA, Chakrapani V, Shunmugam M (2012). Adapting the minority stress model: Associations between gender non-conformity stigma, HIV-related stigma and depression among men who have sex with men in South India. Soc Sci Med.

[25] Graham SM, Micheni M, Kombo B, van der Elst EM, Mugo PM, Kivaya E (2015). Development and pilot testing of the Shikamana intervention to promote care engagement among HIV-positive Kenyan MSM. AIDS.

[26] van der Elst EM, Gichuru E, Muraguri N, Musyoki H, Micheni M, Kombo B (2015). Strengthening healthcare providers’ skills to improve hiv services for men who have sex with men in kenya. AIDS.

[27] Logan CR (1996). Homophobia? No, homoprejudice. J Homosex.

[28] van der Elst EM, Kombo B, Gichuru E, Omar A, Musyoki H, Graham SM (2014). Skills Training of Kenyan Health Care Providers Attending to Men who Have Sex with Men Improved Services Two Years Post Training. AIDS Res Hum Retroviruses.

[29] Sanders EJ, Wahome E, Powers KA, Werner L, Fegan G, Lavreys L (2015). Targeted Screening of At-risk Adults for Acute HIV-1 Infection in Sub-Saharan Africa. AIDS.

[30] Sanders EJ, Wahome E, Mwangome M, Thiong’o AN, Okuku HS, Price MA (2011). Most adults seek urgent healthcare when acquiring HIV-1 and are frequently treated for malaria in coastal Kenya. AIDS.

[31] (2013). The World Factbook 2013-14.

[32] Githuka G, Hladik W, Mwalili S, Cherutich P, Muthui M, Gitonga J (2014). Populations at increased risk for HIV infection in Kenya: results from a national population-based household survey, 2012. J Acquir Immune Defic Syndr.

[33] Gouws E, Cuchi P (2012). International Collaboration on Estimating HIVIbMoT. Focusing the HIV response through estimating the major modes of HIV transmission: a multi-country analysis. Sex Transm Infect.

[34] Bezemer D, Faria NR, Hassan A, Hamers RL, Mutua G, Anzala O (2014). HIV Type 1 transmission networks among men having sex with men and heterosexuals in Kenya. AIDS Res Hum Retroviruses.

[35] Sullivan PS, Carballo-Dieguez A, Coates T, Goodreau SM, McGowan I, Sanders EJ (2012). Successes and challenges of HIV prevention in men who have sex with men. Lancet.

[36] WHO (2015). Guideline on when to start antiretroviral therapy and on pre-exposure prophylaxis for HIV.

